# Retrospective Analysis of Right Iliac Fossa Mass: A Single-Center Study

**DOI:** 10.7759/cureus.27465

**Published:** 2022-07-29

**Authors:** Bharat Kumar Behera, Chandra Sekhar Behera, Manoj Kumar Dehury, Kedar Nath Nayak

**Affiliations:** 1 Department of General Surgery, Government Medical College & Hospital, Sundargarh, IND; 2 Department of Anesthesiology, Saheed Laxman Nayak (SLN) Medical College & Hospital, Koraput, IND; 3 Department of Anatomy, Government Medical College & Hospital, Sundargarh, IND; 4 Department of General Surgery, Bhima Bhoi Medical College & Hospital, Balangir, IND

**Keywords:** tubo-ovarian mass, carcinoma cecum, ileocecal tuberculosis, appendicular lump, right iliac fossa mass

## Abstract

Background

Right iliac fossa mass is one of the most common clinical scenarios a surgeon encounters during their surgical practice. It poses a critical diagnostic dilemma for surgeons and requires considerable diagnostic skills. Its clinical diagnosis becomes difficult in the presence of comorbidities, such as severe obesity and guarding, as in these cases, the mass becomes palpable only upon the administration of a relaxant.

Methods

A total of 108 patients admitted to Bhima Bhoi Medical College & Hospital, exhibiting signs and symptoms of mass in the right iliac fossa upon cross-examination, were included in the study. We recorded their detailed clinical history and performed physical examinations, including erect abdomen, chest (post-anterior view), and contrast x-rays, ultrasonogram; and CT scans.

Results

In this study, patients with appendicular masses, appendicular abscesses, ileocecal tuberculosis, cecum carcinoma, ovarian tumors, and parietal lipoma accounted for 45.3%, 17.5%, 12.9%, 7.4%, 6.4%, and 4.6% of the total cases, respectively, whereas patients with retroperitoneal tumors, parietal abscesses, and ileocecal lymphadenopathy accounted for 1.8% each.

## Introduction

Right Iliac fossa (RIF) mass, referred to as the “temple of surprises”, is a common clinical condition with a notable diagnostic dilemma for surgeons. Most patients diagnosed with a mass in the lower right abdomen are admitted to the surgery ward. The mass can develop from parietal, intra-abdominal, or retroperitoneal structures. The common conditions that present with right iliac fossa mass are appendicular masses, tuberculosis of the ileocecal region, cecal carcinoma, iliac lymphadenitis, and adnexal or tubo-ovarian masses. It is very important to differentiate these conditions to reach a diagnosis and treatment plan as there is vast variability in management. [1]

The aim of the present study is to emphasize the well-recognized clinicopathological aspects of RIF mass in both adolescent and adult populations and its relative incidence in the region, to highlight the mainstay of treatment and its outcome.

## Materials and methods

We systemically examined the hospital database for the diagnoses of RIF mass in a tertiary care hospital in Eastern India, Bhima Bhoi Medical College & Hospital, Balangir, Odisha, between April 2018 and April 2022. A total of 108 patients admitted to the hospital presenting clinical signs and symptoms of RIF mass were included in the study. The criteria for inclusion were patients of both genders of all age groups admitted with a mass in the RIF with or without pain and those patients with incidentally identified RIF mass after examination and investigation. Exclusion criteria were masses developing from other quadrants of the abdomen extending into the RIF, masses arising from structures that are atypically present in the RIF, and swellings arising from adjacent bones.

The detailed clinical history of each patient was recorded, along with a thorough clinical examination. Body parameters, such as complete blood count, erythrocyte sedimentation rate, random/fasting blood sugar, serum urea, creatinine, sodium, and potassium levels were noted. Viral markers for each patient, including the human immunodeficiency virus, hepatitis C virus, and hepatitis B surface antigen were recorded. To verify the diagnosis, ultrasonography (USG) of the abdomen had been advised in all cases. In case of doubtful diagnoses, a contrast-enhanced computed tomography (CECT) scan of the abdomen was advised. In a few cases, colonoscopy was also advised to verify the diagnosis.

Appropriate bowel preparation with suitable antibiotics had been carried out when required. During open laparotomy, all intraabdominal organs were examined, in addition to specific pathological examinations. Surgical procedures were performed according to the type of pathology identified, and postoperative care was provided as per the established protocol. Diagnoses were verified using a histopathological examination of the specimen, and follow-ups with the patients were scheduled at variable time periods.

Data were indicated as frequencies and percentages and analyzed using Microsoft Office Excel 2013 (Microsoft Corporation, Redmond, Washington, United States) and SPSS for Windows, Version 16.0 (Released 2007; SPSS Inc., Chicago, United States). Moreover, a chi-square test was used to determine the association between categorical variables. A p-value less than 0.05 was considered statistically significant.

## Results

This study included 108 patients with masses in the RIF. Data were collated and analyzed and inferences were drawn. Of the total patients, 45.37% were diagnosed with appendicular masses, followed by 17.59% with appendicular abscesses, 12.96% with ileocecal tuberculosis, 7.4% with cecal carcinoma, 6.48% with ovarian tumors, 4.62% with parietal-wall lipoma, and 1.8% patients with retroperitoneal tumors, parietal-wall abscesses, and ileocecal lymphadenopathy each (Table 1).

**Table 1:** Incidence of different types of RIF mass RIF: right Iliac fossa

Serial No.	Diagnosis	No. of cases	Percentage
1	Appendicular lump	49	45.37%
2	Appendicular abscess	19	17.59%
3	Ileocecal tuberculosis	14	12.96%
4	Cecal carcinoma	8	7.4%
5	Tubo-ovarian mass	7	6.48%
6	Parietal lipoma	5	4.62%
7	Retroperitoneal mass	2	1.8%
8	Parietal-wall abscess	2	1.8%
9	Ileocecal lymphadenopathy	2	1.8%
	Total	108	100%

The youngest patient was an 11-year-old boy diagnosed with an appendicular lump and the eldest patient was a 65-year-old diagnosed with cecal carcinoma. Appendicular lump was the most common in the third decade of life, followed by the fourth, fifth, and sixth decades. Additionally, an appendicular abscess was most common in the second decade; tuberculosis of the ileocecal region in the fourth decade; cecal carcinoma in the fifth, sixth, and seventh decades; ovarian tumors and parietal lipomas in the fourth decade; retroperitoneal tumors in the fifth decade; and parietal abscess and iliac lymphadenitis in the third decade of life (Table 2).

**Table 2:** Age distribution

Diagnosis	No. of cases	Second Decade (11–20)	Third Decade (21–30)	Fourth Decade (31–40)	Fifth Decade (41–50)	Sixth Decade (51–60)	Seventh Decade (61–70)
Appendicular lump	49	2	21	7	7	2	0
Appendicular abscess	19	11	5	0	3	0	0
Ileocecal tuberculosis	14	0	4	10	0	0	0
Cecal carcinoma	8	0	0	0	2	4	2
Tubo-ovarian mass	7	2	0	5	0	0	0
Parietal lipoma	5	0	0	4	1	0	0
Retroperitoneal mass	2	0	0	0	2	0	0
Parietal-wall abscess	2	0	2	0	0	0	0
Ileocecal lymphadenopathy	2	0	2	0	0	0	0

Of all diagnoses, appendicular lumps were primarily reported in males (61.22%), whereas appendicular abscesses were mostly reported in females (78.94%). In contrast, tuberculosis of the ileocecal region exhibited equal incidence among males and females. Moreover, cecal carcinoma, parietal lipomas, ileocecal lymphadenitis, and parietal abscess were predominant in males, while retroperitoneal tumors were predominant in females (Table 3).

**Table 3:** Gender composition

Diagnosis	MALE		FEMALE
Appendicular lump	30 (61.22%)	19 (38.77%)	
Appendicular abscess	4 (21.05%)	15 (78.94%)	
Ileocecal tuberculosis	7 ( 50%)	7 (50%)	
Cecal carcinoma	6 (75%)	2 (25%)	
Tubo-ovarian mass	0	7 (100%)	
Parietal lipoma	4 (80%)	1 (205)	
Retroperitoneal mass	0	2 (100%)	
Parietal-wall abscess	2 (100%)	0	
Ileocecal lymphadenopathy	2 (100%)	0	

Patients with appendicular masses (49 cases) predominantly complained of abdominal pain with fever and vomiting in 36 and 32 cases, respectively. Furthermore, the total leucocyte count was high (>11,000) in all patients with appendicular mass and appendicular abscess. USG of the abdomen and pelvis was performed in all cases to confirm the diagnosis. In contrast, in Koch’s total leucocyte count was normal in all 14 cases of ileocecal tuberculosis. Moreover, USG of the abdomen and pelvis and CECT was performed for four patients who tested positive for acid-fast bacilli. Out of eight patients with cecal carcinoma, the total blood count was high in four (Table 4).

**Table 4:** Signs and symptoms

Diagnosis	No. of cases	Fever	Vomiting	Weight loss
Appendicular lump	49	36	32	-
Appendicular abscess	19	19	8	-
Ileocecal tuberculosis	14	7	9	12
Cecal carcinoma	8	2	1	8
Tubo-ovarian mass	7	-	-	4
Parietal lipoma	5	-	-	-
Retroperitoneal mass	2	-	-	-
Parietal-wall abscess	2	2	-	-
Ileocecal lymphadenopathy	2	2	-	1

Conservative management was implemented in patients with an appendicular lump, according to the Ochsner-Sherren regime. Patients who did not respond to conservative management underwent early surgical intervention, with satisfactory post-operative recovery. Out of the 14 patients diagnosed with ileocecal tuberculosis, eight were managed conservatively and six underwent laparotomy and ileotransverse anastomosis. All eight patients were discharged and advised to follow an anti-tuberculosis treatment (ATT) regimen. All eight patients with cecal carcinoma underwent right hemicolectomy and were later referred to a tertiary cancer institute for further treatment (Tables 5, 6).

**Table 5:** Treatment method

Diagnosis	No. of cases	Non-surgical treatment	Surgical treatment
Appendicular lump	49	-	49 (100%)
Appendicular abscess	19	-	19 (100%)
Ileocecal tuberculosis	14	8 (57.14%)	6 (42.85%)
Cecal carcinoma	8	-	8 (100%)
Tubo-ovarian mass	7	-	7 (100%)
Parietal lipoma	5	-	5 (100%)
Retroperitoneal mass	2	-	2 (100%)
Parietal-wall abscess	2	-	2 (100%)
Ileocecal lymphadenopathy	2	2 (100%)	-

**Table 6:** Type of surgical procedure

Surgical procedure	No. of cases	Percentage
Interval appendectomy	30	27.77%
Extra peritoneal drainage with appendectomy	14	12.96%
Right hemicolectomy	8	7.4%
Laparotomy and drainage of abscess	10	9.25%
Right ovarian cystectomy	7	6.48%

## Discussion

The most common condition presenting as RIF mass was appendicular mass, followed by appendicular abscess, Ileocecal tuberculosis, and cecal carcinoma. Similar results have been reported in studies conducted by Juniorsundresh et al. [2] and Raju et al. [3].

Appendicular mass

In the present study, appendicular mass accounted for 45.37% of the cases, with pain being the most common symptom. Fever and vomiting were observed in 73.4% and 65% of patients with appendicular mass, respectively. Moreover, appendicular masses were more common in males than in females (1.57:1). Although only five patients were suspected of a mass in the abdomen, upon examination, a mass in the RIF was confirmed in all cases. According to Das et al., a patient complaining of pain four to seven days after the appearance of symptoms frequently felt a tender mass in the RIF [4].

In this study, all patients of appendicular mass exhibited tender and firm RIF masses. According to Skoubo-Kristensen et al., 55% of the cases in their study exhibited febrile episodes with body temperatures >39 °C [5]. In the present cohort, 73.4% of patients reported fever, whereas vomiting was experienced by 65% of the patients. According to Gahukamble et al., “in situ” delayed appendectomy was beneficial for all patients who responded positively to the initial management of appendicular mass [6].

Skoubo et al. reported that conservative management of appendicular masses was successful in most cases, with lower complication rates than with early operative treatment [5]. Nonetheless, according to Das et al., early appendectomy for the removal of appendicular mass was relatively safe owing to the improvements in surgical techniques and better postoperative care [4]. It also reported that a requirement for prolonged postoperative care was observed in patients in which appendicular mass was managed conservatively compared to patients that underwent early investigations.

Appendicular abscesses were observed in 17.59% of the cohort. Most of the cases were observed in the second decade, and 78.94% of patients were females. According to Bradley et al., the mean age for appendicular abscess formation was 40.7 ± 2.7 years [7].

Ileocecal tuberculosis

According to Elhence et al., although gastrointestinal tuberculosis is rare in developed countries, it is still an issue in developing countries [8]. In this study, 12.96% of the masses in the RIF developed because of tuberculosis. Most cases were reported from rural areas, maximum incidence occurring in the fourth decade (71.4%). The male to female incidence ratio was 1:1, and all patients exhibited abdominal pain, with weight loss reported in 12 cases (85%) and fever in 50% cases. According to Kelly et al., for the diagnosis of Ileocecal tuberculosis, patients with suitable clinical features, even in absence of classical risk factors for tuberculosis, must undergo intense clinical examination [9].

According to Malik et al., USG results can be suggestive of tuberculosis for further diagnosis under proper clinical settings [10]. In the present study, abdominal USG was advised to all patients. A standard drug regimen was prescribed to all patients as per the Revised National Tuberculosis Control Programme (RNTCP) after confirming the diagnosis either by biopsy or after further investigation in the Department of TB & Chest.

Cecal carcinoma

In this study, carcinoma of the cecum was observed in 7.4% of the cases; all patients were above 40 years of age. According to Amin et al., in a study with 20 patients, the majority of the cases were in the age group of 45-65 years, with the oldest patient being 80 years old and the youngest being only 30 years old [11]. In this study, the incidence of cecal carcinoma was higher in males (75%). According to a study by McDermott et al., 55% of patients with cecal carcinoma were males and 49% were females [12].

According to Goligher et al., most of the patients with cecal carcinoma exhibited chronic, but not severe, abdominal pain in the RIF, while the subcostal region or epigastrium was frequently associated with localized tenderness [13]. Richardson et al. reported that abdominal USG findings exhibited a sensitivity, specificity, and accuracy of 96%, 67%, and 91%, respectively, for the diagnosis of colonic carcinoma [14]. In the present study, all patients were accurately diagnosed using USG. Furthermore, a colonoscopy was performed and biopsies were submitted. Considering the growth of ileocecum and ascending colon, Goligher et al. suggested a more extensive right hemicolectomy, unless the general condition of the patient required minimum dissection to ensure a reasonable likelihood of cure [13].

Ovarian tumors

In patients with tubo-ovarian mass, the most common symptoms were mass in the lower right quadrant and loss of weight. The diagnosis was mostly based on USG. In all cases, right ovarian cystectomy was performed, and the excised portion was sent for histopathological examination.

Other patients in this study were diagnosed with parietal lipoma and retroperitoneal mass. In these cases, an excisional biopsy was performed, and the diagnosis was based on histopathological examination. Parietal abscesses were drained externally, while patients with iliac lymphadenopathy, confirmed using USG with guided Fine Needle Aspiration Cytology (FNAC) to be of Koch’s origin, were prescribed drugs under the ATT, as per the RNTCP.

The limitations of the study include being a single-center study with a small sample size.

## Conclusions

In this study, RIF mass was most commonly reported in the age group of 20-40 years, with a higher incidence in males than in females. However, an increased incidence of appendicular abscess was reported in females. The most common symptom was abdominal pain, with pain in the RIF, fever, vomiting, and weight loss among others. Pathology of the vermiform appendix was commonly suspected as a mass in the RIF. Nonetheless, ileocecal tuberculosis was highly suspected in patients with chronic abdominal pain. No mortality was reported in this study. However, surgery was the mainstay of treatment, which presented good outcomes when performed in a proper clinical setting.
